# Trends in Animal Bites and Rabies-related Deaths in Northern Iran: Implications for Public Health Interventions

**DOI:** 10.34172/aim.2024.39

**Published:** 2024-05-01

**Authors:** Ali Davoudi Kiakalayeh, Zakiye Gharib, Reza Mohammadi, Leila Kanafi Vahed, Sajad Davoudi-Kiakalayeh

**Affiliations:** ^1^Department of Preventive and Social Medicine, School of Medicine, Guilan University of Medical Sciences, Rasht, Iran; ^2^Guilan Trauma Institute, Guilan University of Medical Sciences, Rasht, Iran; ^3^Division of Family Medicine and Primary Care, Division of Social Medicine, Department of NVS, Karolinska Institutet, Stockholm, Sweden

**Keywords:** Animal bite, Iran, Public health interventions, Rabies

## Abstract

**Background:**

Rabies remains a public health problem in middle-income countries like Iran, despite being preventable. This study aimed to evaluate the six-year incidence of animal bites in the southern Caspian Sea region from 2016 to 2022, and focus on estimating the direct costs of animal bite cases using the incidence-based method.

**Methods:**

A multicenter, registry-based study was conducted using surveillance data of animal bites.

**Results:**

Of the 40922 cases reported during the study period, 65.9% were male and 34.1% were female. Animal bites were most frequent among individuals over 50 years of age (23.5%), while children under 10 years of age had the lowest frequency of animal bites (2.3%). Animal bites were most common in June. Dogs were responsible for 33277 (81%) cases, cats for 5,624 (13.7%) cases, cows for 1054 (2.5%) cases, and other animals for the remaining cases. During the six-year study period, four deaths due to rabies were reported in the study area. The annual bite incidence rate was 386.3 per 100000 people in northern Iran. The males-to-female ratio was highest in 2019 (M/F ratio=2.4, 95% CI=1.2‒3.4).

**Conclusion:**

The elderly are at higher risk of animal bites, especially in rural areas. It is important to emphasize the use of protective clothing, washing wounds with soap water and rabies vaccination as initial treatment. Targeted vaccination efforts for eligible animals should be prioritized to minimize unnecessary financial burden. Educating farmers about rabies prevention programs, especially in cases of cow bites, is also important.

## Introduction

 Rabies is a neglected viral zoonotic disease in many parts of the world, with over 50 000 deaths occurring annually. To prevent rabies, many people are vaccinated each year in various regions.^1,2^ Previous research indicates that most animal bite patients are children aged 0‒3 years, occur throughout the year, and are predominantly male (males are 1.5 times more likely than females). Rabies is primarily caused by dogs.^3^ It is an endemic disease in Iran, particularly in northern regions close to forests. Rabies is a 100% fatal viral disease that results in acute encephalitis infection of the central nervous system. Currently, there is no drug treatment available for rabies. Despite efforts to combat and quarantine the disease, positive cases still occur in livestock and human populations due to the extensive and varied climate conditions. This leads to economic losses and human casualties, comparable to the economic burden of unintentional accidents on the society.^4,5^ This study presents the new challenges and epidemiological profile of animal bites in the Guilan province, northern Iran, from 2016 to 2022. Our findings could be valuable for low- and middle-income countries.

## Materials and Methods

 This descriptive cross-sectional and multicenter study was conducted to assess the epidemiological profile of animal bite and rabies cases in the Guilan province, northern Iran, from 2016 to 2022. Data on all animal bite cases referred to hospitals, veterinary networks, and health centers in the Guilan province during the study period were collected using a full enumeration method. A checklist was utilized to gather information the characteristics of the bitten individuals, including gender, age, occupation, residential area (urban or rural), and biting animals. The study analyzed information on suspected and confirmed rabies cases, as well as those who received post-exposure prophylaxis (PEP). Descriptive statistics such as frequency and percentage distributions were used to describe the data, while Pearson chi-square test was employed to analyze the research hypothesis and assess the significance of the relationships between variables. All calculations were performed using SPSS version 26, with the significance level set at 0.05 for all tests. Additionally, the study estimated the direct costs of animal bite cases using an incidence-based method, which involved estimating the number of individuals involved in suspected and confirmed rabies cases who received PEP. The direct costs of human diploid cell vaccine (HDCV) for Guilan province in 2022 were calculated based on the reported HDCV doses administrated during the study period and the price per dose. In Iran, each vial of rabies vaccine costs 7€ and each vial of serum (IG) costs €60; all services are free of charge.

## Results

 During the study period, a total of 40 922 animal bites were identified. All cases received outpatient vaccinations. It is worth nothing that only four suspected patients were observed in 2018, none of whom sought PEP at health centers leading to their unfortunate deaths. These four rabies-related deaths included one woman, two men, and a 6-year-old boy, but their data is not included in this study. Over the 6-year study period, in the Guilan province, northern Iran, the annual average of animal bites was 6820 cases, ranging from 4472 in 2016 to 9,891 in 2021. The incidence rate increased from 176.7 per 100 000 population in 2016 to 385.3 per 100 000 population in 2021, showing a consistent upward trend. A significant relationship was observed across the study years, through the Pearson chi-square test (linear-by-linear association). The 95% confidence interval (CI) level was found significant with a *P* value < 0.001. Throughout the study, 65.9% of animal bites were on males and 34.1% were on females, with men consistently at higher risk. Statistical analysis using Pearson chi-square (linear-by-linear association) indicated a significant association between gender and animal bites (*P* < 0.001). The male-to-female ratio was highest in 2019 (M/F ratio = 2.4, 95% CI = 1.2‒3.4) but varied in other years (M/F ratio = 1.31, 95% CI = 0.81‒2.4 in 2016, M/F ratio = 1.95, 95% CI = 1.2‒2.7 in 2017, M/F ratio = 1.5, 95% CI = 0.93‒2.53 in 2018, M/F ratio = 2.3, 95% CI = 0.99‒3.2 in 2020, M/F ratio = 1.98, 95% CI = 1.1‒2.8 in 2021) (Table 1).

**Table 1:** Incidence Rate and Frequency of Suspected Animal Bites Based on Gender in Northern Iran (Guilan Province) from 2016‒2021

**Years**	**Suspected** **Animal Bites (n)**	**Incidence Rate** **(100000opulation)**	**Male-Female (n)**	**Male/Female** **Ratio (95%CI)**
2016	4472	176.7	2526‒1946	1.31 (0.81‒2.4)
2017	5344	211.1	3537‒1807	1.95 (1.2‒2.7)
2018	6258	245.1	3789‒2469	1.5 (0.93‒2.53)
2019	7354	284.7	5215‒2139	2.4 (1.2‒3.4)
2020	7603	295.7	5317‒2286	2.3 (0.99‒3.2)
2021	9891	358.3	6575‒3316	1.98 (1.1‒2.8)

 The frequency of animal bite in the Guilan province from 2016 to 2022, based on the month of the incident, showed an increasing trend from March to September, peaking in May (10%). Most bites occurred in spring and summer months, with a significant association (*P* < 0.001).

 All age groups in the study period saw an increase in rabies-suspected bites, with children under ten accounting for 9.3% and those over 50 being the most frequent victims (23.5%) (Table 2).

**Table 2:** Frequency Distribution of Suspected Animal Bites Based on Age Group in Northern Iran (Guilan Province) from 2016‒2021

**Year**	**1‒4** **No. (%)**	**5‒9** **No. (%)**	**10‒19** **No. (%)**	**20‒29** **No. (%)**	**30‒39** **No. (%)**	**40‒49** **No. (%)**	**+50** **No. (%)**
2016	110 (2.5)	301 (6.7)	582 (13)	821 (18.4)	842 (18.8)	685 (15.3)	1131 (25.3)
2017	127 (2.4)	392 (7.3)	757 (14.2)	1035 (19.4)	933 (17.5)	783 (14.7)	1317 (24.6)
2018	149 (2.4)	486 (7.8)	958 (15.3)	1239 (19.8)	1093 (17.5)	924 (14.8)	1409 (22.5)
2019	169 (2.3)	513 (7)	887 (12.1)	1499 (20.4)	1479 (20.1)	1149 (15.6)	1658 (22.5)
2020	178 (2.3)	523 (6.9)	928 (12.2)	1539 (20.2)	1535 (20.2)	1198 (15.8)	1702 (22.4)
2021	233 (2.4)	618 (6.2)	1278 (12.9)	1875 (19)	1883 (19)	1570 (15.9)	2434 (24.6)

 Regarding animal bites in the Guilan province from 2016 to 2022, domestic animals like dogs (n = 33 277), cats (n = 5624), cows (n = 1054), and others (including horses (n = 108), donkeys (n = 26), sheep (n = 20), mules (n = 7), and goats (n = 6)) were more common than non-domestic animals such as leopards (n = 5), bears (n = 5), bats (n = 7), guinea pigs (n = 37), minks (n = 45), raccoons (n = 30), foxes (n = 17), otters (n = 42), jackals (n = 404), boars (n = 23), wild cats (n = 17), badgers (n = 8), marmots (n = 72), rats (n = 25), monkeys (n = 25), cheetahs (n = 5), and hamsters (n = 35). The trend was significantly increasing over time (*P* < 0.001) (Table 3).

**Table 3:** Animal Involved in Bites in the Guilan Province from 2016 to 2022

**Years**	**Domestic Animal**	**Non-domestic Animal**	**Total**	***P *** **Value**
2016	4389 (88.1)	83 (1.9)	4472	< 0.001
2017	5231 (97.3)	113 (2.7)	5344	
2018	6080 (97.1)	178 (2.9)	6258	
2019	7182 (97.6)	172 (2.4)	7354	
2020	7513 (98.8)	90 (1.2)	7603	
2021	9762 (98.7)	129 (1.3)	9891	
Total	40157 (98.1)	765 (1.9)	40922	

 The study also noted a higher frequency of bites in rural areas compared to urban areas, except in 2022. Cases involving non-vaccinated animals decreased over the study period. The frequency of animal bites varied annually, being highest in 2021 (112 cases,1.47%), and lowest in 2019 (21 cases, 0.3%).

## Discussion

 According to WHO reports, low- and middle-income countries have the highest rate of suspected rabies bites. In the Guilan province, located in northern Iran, the incidence rate of suspected rabies bites is increasing over time. The incidence rate in the study area rose from 176.7 per 100 000 people at baseline in 2016 to 385.3 per 100 000 people at the end of the study in 2021. This increase mirrors the national trend. The study area has a higher rate than the national average, with 385.3 compared to 282 per 100 000 people.^6,7^ The animal bite rate in the study area was similar to those reported in known high-risk populations in lower- and middle-income countries. For example, Mexico reported 273/100 000 people,^8^ and Kenya reported 234/100 000 people.^9^

 When examining the animal bite rates in the study area by age, the findings showed that among 40 922 people with animal bites, 9,651 (23.5%) were in the age group of 50 years and above, indicating the highest frequency of animal bites in this age group. The main reasons for this situation could be the aging of the Iranian society or the higher proportion of elderly people among village residents. The age range of animal bite victims varied widely, from less than one year to 90 years of age. This study supports the findings of previous Iranian studies,^10-12^ that have reported a statistically significant relationship between the age group of patients and the trend of animal bites. However, the most affected age group by animal bites, positive exposure, and rabies deaths in Yemen was 5‒14 years.^13^

 The findings of this study indicate that out of 40 922 examined patients, 26 959 (65.8%) were men, and the remaining were women. Furthermore, there was a significant increasing trend in the number of animal bites every year. The average age of male victims was lower than that of female victims. Men who were victims tended to be either very young or very old. However, some studies have reported that the rate of animal bites is higher among women in certain countries, possibly due to economic and behavioral differences.^14^ For instance, a study conducted in the United States^15^ found that 52.6% of dog bite victims were male and 47.4% were female, while another study conducted in Tehran, Iran, reported that 79.16% of bitten individuals were male.^16^ According to the study results, the incidence rate of suspected rabies bites based on gender varies in different studies and regions. For example, in Turkey, males comprised 66.7% of the cases,^17^ and in Bhutan, there were significantly more bite cases in males (62%) than females (38%).^18^ In Yemen, 66.91% of all cases with rabies exposure were males.^13^

 It is true that compared to women, men are more likely to come into contact with animals which can make them more susceptible to animal bites. Additionally, the higher incidence of animal bites in the spring and summer months can be attributed to the expansion of husbandry and agricultural activities in the region. Similar findings have been reported in studies conducted in different regions of Iran,^19^ with an upward trend observed for animal bite cases over time. This study suggests that appropriate intervention programs are necessary to prevent and reduce the incidence of animal bites, particularly rabies, and that education and awareness campaigns should target high-risk groups such as young people and rural populations. The incidence of animal bites is higher in the spring and summer seasons due to increased outdoor activities and exposure to animals. However, it is important to note that animal bites can occur at any time of the year and in any location, not just in rural areas. Therefore, individuals should take precautions when interacting with animals, such as avoiding contact with unfamiliar animals, properly securing garbage and food sources, and seeking medical attention immediately if bitten. Additionally, vaccination programs for domestic animals, such as dogs and cats, can also help reduce the incidence of animal bites and the spread of diseases like rabies. Overall, a combination of education, awareness, and intervention programs can help prevent and reduce the incidence of animal bites and promote public health and safety.

 Based on the results of this study, the frequency of animal bites by domestic animals was higher than that of non-domestic animals in the study area. Out of 49 500 cases, 40 157 (81.3%) were bitten by domestic animals. A study conducted in Birjand, Iran, reported that 86.3% of animal bites were caused by domestic animals, with most cases involving dogs. Similar findings have been reported in studies conducted in different regions of Iran, with an upward trend observed for animal bite cases over time. However, there was a significant relationship between the biting animal and the occurrence of the accident.^12,19^

 On the other hand, another study conducted in Iran observed that most cases of animal bites were caused by non-domesticated animals, which differs from the findings of this study. In the present study, the highest frequency of bites occurred by dogs, accounting for 81.3% of cases, followed by cats with 13.7% of cases. Animal bites caused by cows ranked third, comprising 2.5% of cases. The two reasons for the increasing cases of animal bites by cows are the development of buildings in villages where jackals reside, leading to the transmission of rabies from jackals to cattle, and farmers’ lack of familiarity with rabies symptoms in cattle. Farmer may also mistakenly assume that cattle are exhibiting symptoms of consuming plastic garbage, leading them to attempts to remove it from the animal’s mouth and subsequently getting bitten. Previous studies have confirmed that the highest number of animal bites were from dogs followed by cats.^3,20^ However, the present study is the first to show the magnitude of the prevalence of animal bites by cows in Iran. Regarding the type of attacking animal, our study shows that dogs are the most common attacking animal in Iran, consistent with the findings of other studies.^21^ Dogs were the attacking animal in 91.3% of animal bite cases during the study period. Most studies have reported that dogs are the most significant cause of human bites. Therefore, the priority of the bite control and prevention program should focus on reducing dog bites. Increasing community awareness and knowledge about how to deal with dogs, such as not leaving herds and domestic dogs unattended, collaring or sterilizing dogs on the outskirts of cities and villages, identifying high-risk areas, and implementing special planning for those areas, can reduce dog bite cases. The present study shows that dogs are the most common attacking animal in Iran, and the frequency of animal bites was higher in villages than in cities, with a significant increasing trend over time.

 However, in 2021, the animal bite cases in urban areas was slightly higher than rural areas. Two reasons can be put forward for the increase in animal bites in cities in Iran. One is the issue of stray animals, whose numbers are increasing in cities, and the other reason is the increase in keeping dogs and cats in cities. One study^10^ showed that most cases of animal bites occurred in urban residents, but there was no statistically significant correlation between people’s place of residence and the occurrence of animal bites. However, the present study found that the rate of animal biting by dogs in rural areas was statistically higher than that of urban residents, which is consistent with the findings of Sabouri et al^3^ In addition to the measures mentioned above, it is essential to promote responsible pet ownership, including proper training and socialization of dogs, to minimize the risk of dog bites. Educating the public on how to interact with dogs safely and recognize signs of aggression can also help prevent incidents. Furthermore, implementing and enforcing strict animal control laws, such as leash laws and regulations on breeding and selling dogs, can contribute to reducing the number of dog bites. Collaboration between local authorities, veterinarians, and animal welfare organizations is crucial for the success of these initiatives. Lastly, providing accessible and affordable rabies vaccination programs for dogs can help control the spread of the disease and reduce the public health impact of dog bites. By addressing the issue of dog bites comprehensively and proactively, it is possible to reduce the number of incidents and improve overall public safety in both urban and rural areas.

 Based on the results obtained from the history of bites, the frequencies were different in the years under review, with the highest frequency observed in 2020 and the lowest frequency observed in 2018. Vaccination played an important role in reducing the incidence of rabies in the animal bite process, which was in line with the findings of our study. The study showed that all patients recovered completely. The trend of changes in animal bites among animals not subject to rabies vaccination was decreasing over time in the present study. During the study period, it decreased from 372 cases to 106 cases. Vaccination had an important role in reducing the incidence of rabies in the animal bite process, which was consistent with other studies conducted in Iran.^22,23^ The search results suggest that vaccination of dogs and prevention of dog bites can prevent rabies. After a potential exposure of people to a rabid animal, a fast-acting shot (rabies immune globulin) can prevent the virus from infecting the person.

 Regarding the outcome of patients, it was found that all patients recovered and were discharged, and no deaths due to animal bites were reported during the six years under study, which is consistent with recent study results.^8^ Although no deaths due to animal bites after vaccination were reported during the six-year study, the costs incurred for the health system due to vaccination are higher than the costs of preventing other accidents in the same region.^24,25^

## Conclusion

 Preventing animal bites requires a combination of education, awareness, and access to proper medical treatment. The elderly are at higher risk of animal bites, especially in rural areas. It is important to emphasize the use of protective clothing and washing wounds with soap and water as the initial treatment. Immunizing animals, especially dogs, is crucial in preventing bites. Immediate and timely referral to health centers and anti-rabies vaccination is essential after a bite to prevent death. Bats and raccoons can carry the rabies virus, so seeking medical attention after a bite from an animal unknown or wild is crucial. Controlling rabies in domestic animals through vaccination is the most effective prevention measure. Spaying and neutering pets and keeping them supervised can reduce risk. Targeted vaccination efforts for eligible animals should be prioritized to minimize unnecessary financial burden. Educating farmers about rabies prevention programs, especially in cases of cow bites, is also important.
